# The impact of the severity of sepsis on the risk of hypoglycaemia and glycaemic variability

**DOI:** 10.1186/cc7097

**Published:** 2008-10-21

**Authors:** Reiner M Waeschle, Onnen Moerer, Reinhard Hilgers, Peter Herrmann, Peter Neumann, Michael Quintel

**Affiliations:** 1 Department of Anaesthesiology, Emergency and Intensive Care Medicine, University of Goettingen, Robert-Koch-Strasse 40, Goettingen, 37075, Germany; 2 Department of Medical Statistics, University of Goettingen, Humboldtallee 32, Goettingen, 37073, Germany

## Abstract

**Introduction:**

The purpose of this study was to assess the relation between glycaemic control and the severity of sepsis in a cohort of patients treated with intensive insulin therapy (IIT).

**Methods:**

In a prospective, observational study, all patients in the intensive care unit (ICU) (n = 191) with sepsis, severe sepsis or septic shock were treated with IIT (target blood glucose (BG) level 80 to 140 mg/dl instead of strict normoglycaemia). BG values were analysed by calculating mean values, rate of BG values within different ranges, rate of patients experiencing BG values within different levels and standard deviation (SD) of BG values as an index of glycaemic variability.

**Results:**

The number of patients with hypoglycaemia and hyperglycaemia was highly dependent on the severity of sepsis (critical hypoglycaemia ≤ 40 mg/dl: sepsis: 2.1%, severe sepsis: 6.0%, septic shock: 11.5%, p = 0.1497; hyperglycaemia: >140 mg/dl: sepsis: 76.6%, severe sepsis: 88.0%, septic shock: 100%, p = 0.0006; >179 mg/dl: sepsis: 55.3%, severe sepsis: 73.5%, septic shock: 88.5%, p = 0.0005; >240 mg/dl: sepsis: 17.0%, severe sepsis: 48.2%, septic shock: 45.9%, p = 0.0011). Multivariate analyses showed a significant association of SD levels with critical hypoglycaemia especially for patients in septic shock (p = 0.0197). In addition, SD levels above 20 mg/dl were associated with a significantly higher mortality rate relative to those with SD levels below 20 mg/dl (24% versus 2.5%, p = 0.0195).

**Conclusions:**

Patients with severe sepsis and septic shock who were given IIT had a high risk of hypoglycaemia and hyperglycaemia. Among these patients even with a higher target BG level, IIT mandates an increased awareness of the occurrence of critical hypoglycaemia, which is related to the severity of the septic episode.

## Introduction

Hyperglycaemia and insulin resistance are common in patients in the intensive care unit (ICU) and are associated with a substantial increase in mortality [1,2]. Major causes of morbidity and death include severe infection, critical illness polyneuropathy and multi-organ failure [3]. Intensive insulin therapy (IIT) aimed at achieving a blood glucose (BG) level between 80 and 110 mg/dl was shown to decrease morbidity (eg, reduced severe infections and organ failure) and mortality in adult surgical ICU patients [3]. In medical ICU patients, the mortality rate was only decreased in the subgroup of patients that stayed in the ICU for three or more days [4].

Severe sepsis and septic shock are major causes of mortality in ICU patients [5,6]. The impact and safety of IIT for these patients is controversial. The Surviving Sepsis Campaign Guidelines recommend a target BG level below 150 mg/dl [7]. One of the most commonly mentioned and feared adverse effects of IIT is severe hypoglycaemia, which occurs significantly more often when using IIT compared with conventional insulin therapy [3,4,8]. The German multicentre Efficacy of Volume Substitution and Insulin Therapy in Severe Sepsis (VISEP) study of IIT for septic patients in multidisciplinary ICUs was prematurely stopped due to a higher risk of hypoglycaemia using IIT compared with conventional insulin therapy (17.0% versus 4.1%) [8].

Analysis of the actual BG levels of septic patients with target BG levels between 80 and 120 mg/dl revealed an increased risk of hypoglycaemia (BG levels < 80 mg/dl) [9]. Recently, predisposing factors for hypoglycaemia in a mixed medical/surgical ICU population were identified; the risk factors include insulin use, sepsis, continuous renal replacement therapy (CRRT) with bicarbonate-based substitution fluid, diabetes, nutrition decrease without adjustment for insulin infusion and inotropic support [10,11]. Previous studies have not differentiated between severe sepsis and septic shock, and they have used only the mean (morning) BG level per day as the parameter to evaluate BG dynamics. Therefore, it is unknown if the severity of sepsis is directly associated with a higher risk of hypoglycaemia and if analysis of the mean BG level per day is sufficient and appropriate for the analysis of BG dynamics.

The aim of this study was to analyse the quality of BG control using different parameters and the risk for critical hypoglycaemia (≤ 40 mg/dl) in patients with sepsis, severe sepsis and septic shock receiving IIT with a target BG level of 80 to 140 mg/dl. In addition, potential predisposing factors for hypoglycaemia were analysed.

## Materials and methods

### Study population

The 'closed' 42-bed ICU at the University hospital of Goettingen receives patients mainly from surgical departments, but also from medical and neurology units. Most patients are admitted because of cardiac, neurological, abdominal or trauma-related surgery. The nurse:patient ratio ranges from 1:2 to 1:3. The local ethics committee approved the study and waived patients' informed consent. Data collection began after an eight-week introduction period. All patients admitted from July to September 2004 or from November to December 2004 (n = 191) and who fulfilled the criteria of sepsis either on admission or at any time during their ICU stay were included in the study. They were sequentially treated with one of two different nurse-driven IIT protocols for four months. The assignment to a specific protocol depended on the date of admission. No randomisation was performed. The study design was strictly observational, and the members of our study group who performed data assessment were completely separate from the regular ICU staff.

### Sepsis data assessment and analysis

The assessment of sepsis severity was retrospectively performed day by day according to the classification criteria set forth by the German Sepsis Society [12] by one investigator (RW) after a two-month training period. Septic patients were assigned to one of the following three categories after discharge from the ICU according to the most severe degree of sepsis observed during the entire ICU stay.

Sepsis was defined as the presence of two or more of the following systemic inflammatory response syndrome (SIRS) criteria associated with infection: tachycardia (≥ 90 beats/minute); tachypnoea (≥ 20 breaths/minute) or hyperventilation (pCO_2 _≤ 4.3 kPa/≤ 33 mmHg); leucocytosis (≥ 12,000 cells/mm^3^), leucocytopenia (≤ 4000 cells/mm^3^) or normal white blood cell (WBC) count with 10% of more immature cells; and fever (core temperature ≥ 38.0°C) or hypothermia (core temperature ≤ 36.0°C) [12].

Severe sepsis was defined as secondary organ failure due to sepsis. Criteria for secondary organ failure were: acute encephalopathy, relative or absolute thrombocytopenia (≤ 100,000 platelets/mm^3 ^or reduction of more than 30% within 24 hours), arterial hypoxaemia (PaO_2 _≤ 10 kPa/≤ 75 mmHg on natural air or PaO_2_/FiO_2 _≤ 33 kPa/≤ 250 mmHg), impaired renal function (urine output ≤ 0.5 ml/kg/hour or serum creatinine more than twice normal value) or metabolic acidosis (base excess ≤ -5 or hyperlactataemia more than 1.5 times normal value).

Septic shock was defined as persistent hypotension (systolic BP < 90 mmHg/mean BP < 65 mmHg) unresponsive to volume resuscitation with the need for vasopressor therapy [12].

The treatment of sepsis was carried out according to the guidelines of the Surviving Sepsis Campaign [7].

In addition, all patient data, including vital parameters and laboratory results, were collected in the electronic patient data management system (PDMS) of the ICU. Furthermore, parameters defining SIRS [13] and secondary organ failure, the assessment of infectious status, as well as Simplified Acute Physiology Score (SAPS) II [14] and Sepsis-Related Organ Failure Assessment (SOFA) [15] scores were prospectively collected on a daily basis and processed in an Access database, Office 2003, Microsoft, USA.

### Intensive insulin therapy

The target BG level for both therapy protocols was 80 to 140 mg/dl. This higher range compared with the study protocol of Van den Berghe and colleagues [3,4] and was chosen because of the concern for hypoglycaemia in a 'real life', non-study setting. Protocol 1 was a 'sliding scale' protocol with a combination of continuous insulin infusion (50 IU Actrapid in 50 ml of 0.9% sodium chloride using an infusion pump (Braun, Germany)) and an additional IV bolus application using a specified treatment algorithm based on the actual glucose value (additional file 1). Using a sliding scale, a predefined amount of insulin was given according to the range of the actual BG value [16].

Protocol 2 was a 'dynamic scale' protocol with continuous insulin infusion (without any bolus application) based on the protocol of Kanji and colleagues [17] (additional file 2). Using a dynamic scale, the dosage of the continuous insulin infusion was increased or decreased by an amount that was dependent on the range of the previous BG value compared with the actual BG level [16]. It is important to note that the second protocol did not include a bolus insulin application and the rate of continuous insulin infusion was not limited.

In all patients, a balanced full electrolyte crystalloid solution containing 5% glucose (1 mg/kg/hour Sterofundin BG-5 (Braun, Germany)) was started on admission. Enteral or parenteral nutrition was started on day two after admission. Nutritional support was not further controlled by our study protocol; it was prescribed by the attending physicians. BG levels were measured predominantly in arterial blood on admission and at least every three hours thereafter (every 30 to 60 minutes in the case of hypoglycaemic values; if an arterial line was not in place, central venous blood was sampled) using an ABL 700 blood sample analyser (Radiometer Medical, Copenhagen).

### Blood glucose level

The quality of BG control by IIT was evaluated measurement of the overall mean BG level; the morning BG level; the number of patients who had at least one BG value within indicated BG ranges (hypoglycaemia ≤ 40 mg/dl, 41 to 60 mg/dl, 61 to 79 mg/dl, normoglycaemia 80 to 40 mg/dl and hyperglycaemia 141 to 179 mg/dl, 180 to 239 mg/dl and ≥ 240 mg/dl); the number of critical hypoglycaemic episodes (≤ 40 mg/dl) and the rate of those episodes per 100 hours of IIT. In addition, the rate of BG values within different levels, which were divided into the indicated groups, and the median number of BG measurements obtained per day were analyed. As an index of glycaemic variability, we calculated the standard deviation (SD) of all BG values for each patient. The time to reach the BG target was defined as the interval from the first insulin application to the first BG value inside the target range. The incidence of critical hypoglycaemia (≤ 40 mg/dl) was compared during days with and without sepsis. In addition, the time interval from the previous BG measurement to the following critical hypoglycaemic episode itself and the time intervals between all BG measurements within the 24 hours before a hypoglycaemic episode were analysed. All analyses were performed separately for septic episodes; for the entire ICU stay; for each IIT protocol; and for the groups stratified by the severity of sepsis.

### Insulin therapy

The duration of continuous insulin application, the duration of overall insulin therapy (interval from the first to the last insulin application), and the given insulin dose per day were compared.

### Morbidity and mortality

In order to assess morbidity and mortality, the following factors were analysed: the rate of acute renal failure, rate of patients with CRRT (using primarily bicarbonate-based replacement fluids), frequency of readmission to the ICU during the same hospital admission, duration of the septic episode for the groups stratified by the severity of sepsis, length of mechanical ventilation, length of stay (LOS), and mean SAPS II and SOFA scores for each patient. In addition, 28-day, ICU and hospital mortality rates were analysed. Acute renal failure was defined as a creatinine level of more than twice the normal upper limit, an increase of creatinine to twice the value on admission, and/or acute oliguria for at least two hours (urine output < 0.5 ml/kg/hour). IIT seems to improve the mortality rates of patients with prolonged ICU stays [3,4], so the 28-day and ICU mortality rates were analysed separately for patients with a LOS of more than three days and more than five days.

### Predisposing factors for hypoglycaemia

We studied the following possible predisposing factors for critical hypoglycaemia (BG level ≤ 40 mg/dl): age, gender, type of admission (medical, scheduled surgery and unscheduled surgery), IIT protocol, SAPS II and SOFA scores on the first day of sepsis, history of diabetes, acute renal failure, CRRT, inotropic/vasopressor therapy, hydrocortisone therapy, LOS and duration of ventilation.

### Statistical analysis

Pearson chi-squares 2 × 2 contingency table, Mann-Whitney U test and student's t-test (to compare parameters between the different treatment groups) or a chi-squares test, Kruskal–Wallis analysis of variance (ANOVA) and univariate ANOVA (to compare parameters within the different sepsis level groups) were used as appropriate. To define predisposing factors, chi-squared tests were used for categorical parameters and odds ratios (OR) and 95% confidence intervals (CI) were used for binary parameters. For single parameters, mean/SD or median/interquartile range (IQR) values are given. Multivariable logistic regression analyses were performed using advanced linear/nonlinear models.

We created a model to predict mortality, including rate of critical hypoglycaemia (≤40 mg/dl), median SD of BG levels for each patient, IIT protocol and a model to predict the occurrence of severe hypoglycaemia, including median SD of BG levels, severity of sepsis, IIT protocol, history of diabetes, CRRT, catecholamine therapy, mechanical ventilation, rate of critical hypoglycaemia and mortality. The significance level was set at p = 0.05. All analyses were performed using the Statistica 8.0 software (StatSoft Inc., USA).

## Results

### Comparison of the severity of sepsis

Table 1 shows the baseline characteristics according to the severity of sepsis and table 2 shows the corresponding outcome parameters. The differentiation into the sepsis groups led to a significant distinction of outcome parameters.

**Table 1:** Baseline characteristics for different severities of sepsis

**Baseline characteristics**	**Sepsis n = 47**	**Severe sepsis n = 83**	**Septic shock n = 61**	**p values**^a^
Age (median years, IQR)	68.0 (47 to 75)	70.0 (58 to 77)	65.0 (54 to 74)	0.1152^c^
Gender (male; %/n)	63.8% (30)	61.4% (51)	67.2% (41)	0.7761^b^
BMI (kg/m^2^; median/IQR)	25.7 (22.9 to 29.4)	25.9 (23.1 to 29.0)	27.2 (24.5 to 31.2)	0.0787^c^
Initial GCS^d ^(median/IQR)	15 (14 to 15)	15 (15 to 15)	15 (12 to 15)	0.2473^c^
Readmission (%/n)	12.8% (9)	21.7% (18)	14.8% (9)	0.3552^b^
Reason for admission (%/n)				
- Medical	17.0% (8)	25.3% (21)	31.1% (19)	
- Scheduled surgical	31.9% (15)	27.7% (23)	24.6% (15)	0.5774^b^
- Unscheduled surgical	51.1% (24)	47.0% (39)	44.3% (27)	
Initial SAPS II [16] (median/IQR)	36.5 (32.0 to 44.0)	41.5 (33.0 to 48.0)	43.5 (36.5 to 51.0)	**0.0453**^c^
Initial SOFA [17] (median/IQR)	8.0 (5.0 to 9.0)	7.5 (5.0 to 10.0)	9.0 (7.0 to 11.0)	**0.0004**^c^
Predicted mortality rate (by SAPS II) (%; median/IQR)	18.9 (12.8 to 32.6)	27.6 (14.0 to 41.5)	31.6 (18.9 to 48.4)	**0.0453**^b^
Initial blood glucose^e ^(mg/dl; median/IQR)	130.5 (110.5 to 161.5)	150.0 (125.5 to 203.0)	144.0 (115.0 to 173.0)	0.1403^c^
History of diabetes (%/n)	17.0% (8)	39.8% (33)	23.7% (14)	**0.0148**^b^
Acute renal failure, preadmission (n/%)	0.0% (0)	4.8% (4)	11.9% (7)	**0.0265**^b^

^a ^Significance level p < 0.05; p values for the comparison between the different sepsis groups were calculated using Chi-square test (b) and Kruskal–Wallis analysis of variance (c), as appropriate. ^e ^To convert the values for glucose from mmol/L to mg/dl, multiply by 18.018.

BMI = Body Mass Index; GCS = Glasgow Coma Scale; IQR = Interquartile Range; SAPS II = Simplified Acute Physiology Score; SOFA = Sepsis-Related Organ Failure Assessment.

**Table 2:** Outcome parameters for different severities of sepsis

**Outcome parameters**	**Sepsis n = 47**	**Severe sepsis n = 83**	**Septic shock n = 61**	**p values**^a^
Median SAPS II [14] (median/IQR)	38.1 (28.1 to 42.0)	38.0 (33.5 to 44.0)	45.8 (38.3 to 51.5)	**< 0.0001**^b^
Median SOFA [15] (median/IQR)	6.0 (4.0 to 7.6)	5.5 (4.5 to 7.5)	8.9 (7.0 to 11.3)	**< 0.0001**^b^
Acute renal failure, post-admission (n/%)	10 (21.3%)	37 (44.6%)	34 (55.7%)	**0.0008**^c^
Patients with CRRT (n/%)	6 (12.8%)	12 (14.5%)	21 (35.0%)	**0.0035**^c^
Duration of septic episodes (days; median/IQR)	2.0 (1.0 to 6.0)	4.0 (2.0 to 7.0)	10.0 (7.0 to 19.0)	**<0.0001**^b^
Time of ventilation (hours; median/IQR)	37.3 (0.0 to 760.9)	61.0 (0.0 to 933.9)	308.1 (2.4 to 2052.9)	**< 0.0001**^c^
Length of stay (hours; median/IQR)	166.5 (95.0 to 320.0)	213.0 (135.0 to 412.0)	391.0 (216.0 to 870.0)	**< 0.0001**^c^
28 day mortality (n/%)	5 (10.6%)	7 (8.4%)	15 (24.6%)	**0.0166**^b^
ICU mortality (n/%)	5 (10.6%)	9 (10.8%)	25 (41.0%)	**< 0.0001**^b^
Hospital mortality (n/%)	7 (15.6%)	13 (16.5%)	26 (43.3%)	**0.0003**^b^

^a ^Significance level p < 0.05; p values for the comparisons between the different sepsis groups were calculated using Kruskal–Wallis analysis of variance (b) and Chi-square test (c), as appropriate.

CRRT = continuous renal replacement therapy; ICU = intensive care unit; IQR = Interquartile Range; SAPS II = Simplified Acute Physiology Score; SOFA = Sepsis-Related Organ Failure Assessment.

**Figure 1:**
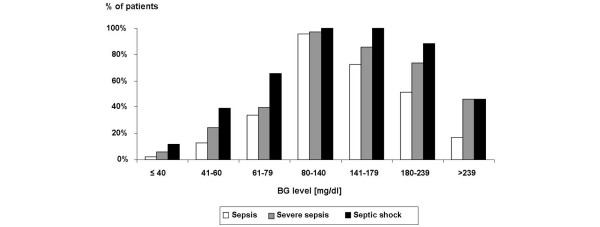
**Rate of patients with the indicated blood glucose (BG) levels during sepsis across the different sepsis groups**. The different columns correspond to the proportion of patients who had at least one BG value within the indicated BG ranges during sepsis. Note that a patient can be included in more than one column.

The rate of patients with critical hypoglycaemia (≤ 40 mg/dl) tended to be higher in the group of patients who developed severe sepsis, especially septic shock, during septic episodes (sepsis: 2.1%, severe sepsis: 6.0%, septic shock: 11.5%, p = 0.1497, Figure 1); however, this association did not hold true when the entire ICU stay was analysed (sepsis: 12.8%, severe sepsis: 14.5%, septic shock: 13.1%, p = 0.9551). Figure 1 shows the percentage of patients who had at least one BG value within the indicated BG ranges across the different sepsis groups.

**Figure 2:**
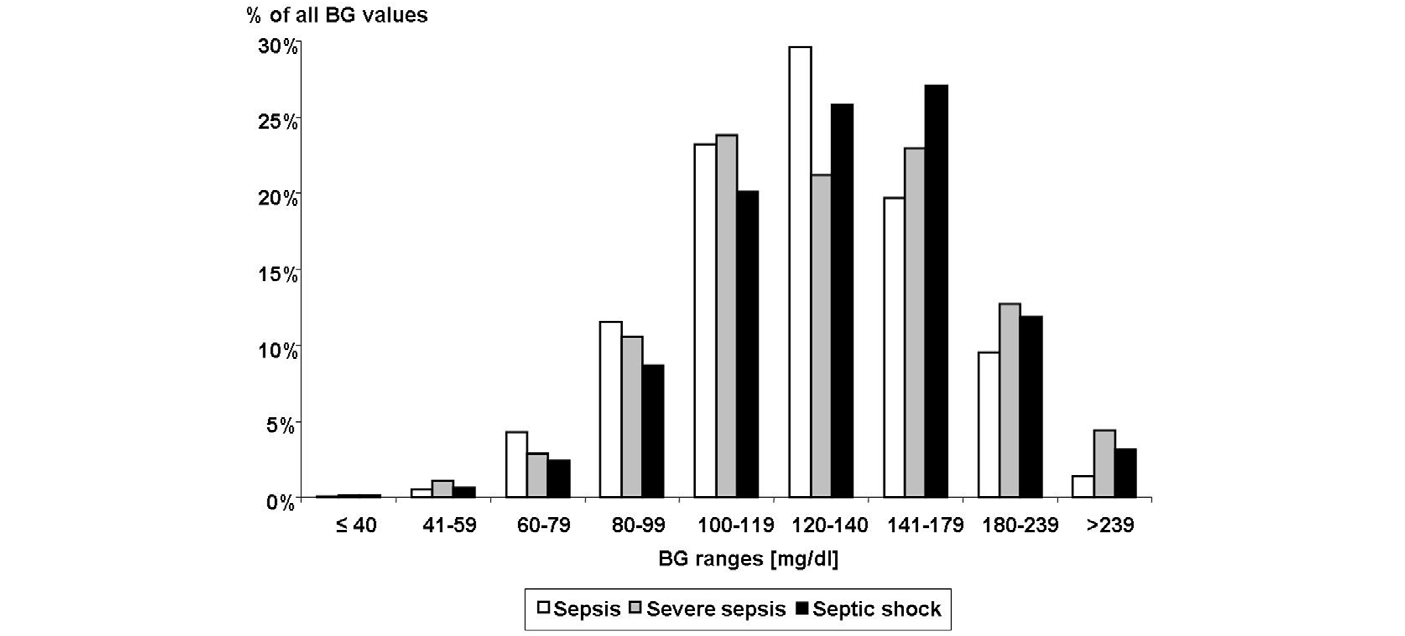
**Distribution of blood glucose (BG) values across the different sepsis groups**. Each column of the histogram represents the portion of BG values relative to all measured values in the different BG ranges: hypoglycaemia ≤ 40 mg/dl, 41 to 59 mg/dl, 60 to 79 mg/dl; normoglycaemia 80 to 99 mg/dl and 100 to 140 mg/dl; and hyperglycaemia 141 to 179 mg/dl, 180 to 239 mg/dl, ≥ 240 mg/dl.

The rate of hyperglycaemic BG values was also higher for patients with severe sepsis and septic shock compared with patients with sepsis (rate of BG values > 140 mg/dl: sepsis: 30.8%, severe sepsis: 40.2%, septic shock: 41.9%, p < 0.0001). Figure 2 details the distribution of all measured BG values in the following categories: hypoglycaemia ≤ 40 mg/dl, 41 to 59 mg/dl, 60 to 79 mg/dl; normoglycaemia 80 to 99 mg/dl, 100 to 119 mg/dl, 120 to 140 mg/dl; and hyperglycaemia 141 to 179 mg/dl, 180 to 239 mg/dl, ≥ 240 mg/dl). The SD of all measured BG values for each patient was significantly higher for patients with severe sepsis relative to the other groups with sepsis and septic shock (SD median (IQR): sepsis: 29.5 mg/dl (19.2 to 35.9 mg/dl), severe sepsis: 38.5 mg/dl (26.0 to 52.1 mg/dl), septic shock: 31.1 mg/dl (25.2 to 46.9 mg/dl); p = 0.0090).

**Figure 3:**
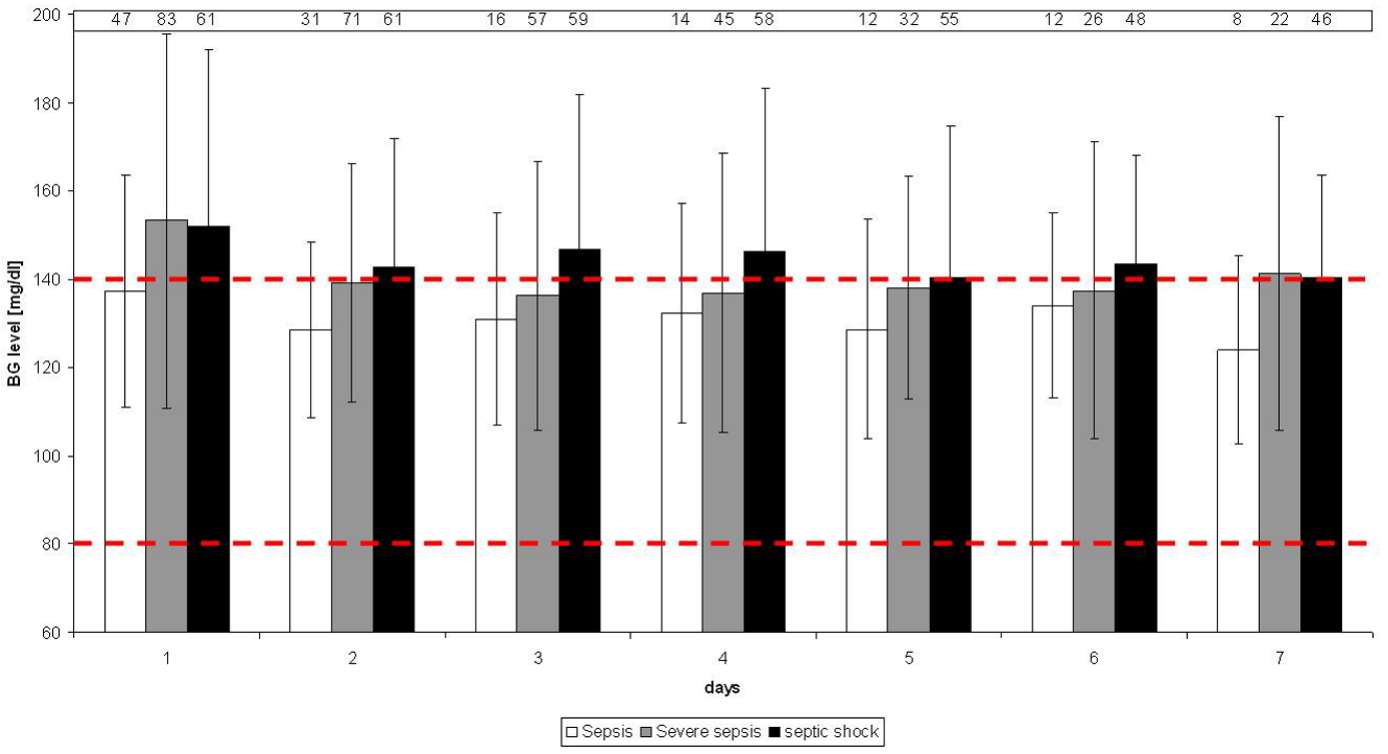
**Mean blood glucose (BG) level per day over time for the different sepsis subgroups**. Each column represents the mean BG level of all patients during sepsis. The different levels of sepsis are grouped for each of the first seven days. The number above each error bar indicates the number of patients.

Table 3 shows the median number of BG measurements collected per day and the number of hypoglycaemic episodes. Patients with critical hypoglycaemia during sepsis also had a significantly higher risk of critical hypoglycaemia outside of septic episodes (hypoglycaemic patients during sepsis: 30.8% of patients with hypoglycaemia outside sepsis, non-hypoglycaemic patients during sepsis: 8.3% of patients with hypoglycaemia outside sepsis, p = 0.0323). Table 3 lists the different time intervals from BG measurements to each hypoglycaemic episode. Figure 3 details the mean BG levels measured per day.

**Table 3:** Analyses of blood glucose (BG) levels and insulin therapy across groups with different severities of sepsis

**BG and insulin analyses**	**Sepsis n = 47**	**Severe sepsis n = 83**	**Septic shock n = 61**	**p values**^a^
Mean BG level during sepsis (mg/dl; mean/SD)	132.0 (21.4)	142.4 (25.7)	140.2 (21.4)	0.0516^b^
Median BG level during sepsis (mg/dl; median/IQR)	133.7 (113.5 to 142.1)	142.1 (122.5 to 158.3)	133.5 (124.0 to 161.3)	**0.0458**^c^
Mean morning BG level during sepsis (mg/dl; mean/SD)	124.4 (17.5)	131.0 (± 32.5)	135.1 (54.4)	0.7227^b^
Median SD during sepsis (mg/dl; median/IQR)	29.5 (19.2 to 35.9)	38.5 (26.0 to 52.1)	31.1 (25.2 to 46.9)	**0.0090**^c^
Median morning BG level during sepsis (mg/dl; median/IQR)	126.3 (113.0 to 136.0)	122.5 (107.2 to 152.0)	121.8 (109.0 to 139.5)	0.9552^c^
Median number of BG measurements per day during sepsis (median/IQR)	7.0 (5.6 to 8.5)	7.0 (5.5 to 8.5)	8.7 (7.6 to 9.7)	**0.0001**^c^
Absolute number of critical hypoglycaemic episodes *	1	6	7	
Rate of critical hypoglycaemia per 100 hours of IIT (median/IQR)	0.17 (single episode)	0.81 (0.48 to 1.01)	0.37 (0.16 to 0.58)	0.3278^c^
Median time interval of BG measurements within 24 hour prior to the critical hypoglycaemic episode (hours; median/IQR)	4.1 (3.6 to 5.2)	2.0 (1.5 to 2.3)	2.5 (1.5 to 2.8)	0.1715^c^
Median time interval from the last BG measurement before to the critical hypoglycaemic episode itself (hours; median/IQR)	3.9 (single episode)	1.4 (0.7 to 3.1)	2.4 (1.3 to 4.3)	0.5619^c^

* The median number of episodes per patients was 1 in each category.

^a ^Significance level p < 0.05; p values for comparisons between the different sepsis groups were calculated using ANOVA (b) and Kruskal–Wallis analysis of variance (c), as appropriate.

IQR = interquartile range; IIT = intensive insulin therapy; SD = standard deviation.

Patients with septic shock had an increased duration of continuous and overall insulin therapy (duration of continuous therapy in median (IQR): sepsis: 83.4 hours (50.3 to 166.4 hours), severe sepsis: 101.1 hours (18.4 to 199.7 hours), septic shock: 217.6 hours (110.8 to 466.1 hours), p = 0.0001; duration of overall insulin therapy: sepsis: 134.0 hours (72.1 to 251.4 hours), severe sepsis: 184.3 hours (93.7 to 310.7 hours), septic shock: 320.2 hours (158.0 to 789.9 hours), p < 0.0001). Similarly, the median insulin dose per day applied over the entire ICU stay was higher for patients with septic shock (median (IQR) insulin dose per day: sepsis: 33.2 IU (22.7 to 47.6 IU), severe sepsis: 36.3 IU (21.4 to 48.7 IU), septic shock: 45.0 IU (34.6 to 68.9 IU), p = 0.0005).

### Comparison of the two IIT protocols

No differences were observed when comparing baseline characteristics and outcome parameters, with the exception of age (median years; protocol 1: 67.0, protocol 2: 70.0, p = 0.0336) and gender (female; protocol 1: 41.0%, protocol 2: 27.5%, p = 0.0496). Table 4 shows further comparisons between the two protocols. Additional file 2 details the mean BG levels per day.

**Table 4:** Comparison between the two intensive insulin therapy (IIT) protocols

**Comparison of the two IIT protocols**	**Protocol 1 (n = 100)**	**Protocol 2 (n = 91)**	**p values**^a^
Mean BG level during sepsis (mg/dl; mean/SD)	145.9 (24.7)	131.7 (19.9)	**<0.0001**^b^
Median amount of insulin per day (IU; median/IQR)	42.8 (29.6 to 68.4)	38.6 (24.4 to 58.3)	0.2165^c^
Median duration of continuous insulin therapy (hours; median/IQR)	102.2 (8.2 to 214.2)	143.6 (63.8 to 297.7)	**0.0058**^c^
Median duration of entire insulin therapy (hours; median/IQR)	188.0 (101.9 to 431.5)	210.4 (103.9 to 429.6)	0.7102^c^
Incidence of hyperglycaemic values	49.7%	32.3%	**<0.0001**^d^
Frequency of BG values within the target range	47.7%	63.2%	**<0.0001**^d^
Time to reach target BG level (hours; median/IQR)	7.7 (3.2 to 34.8)	4.1 (3.2 to 09.01)	**0.0012**^c^
Duration of continuous insulin therapy (hours; median/IQR)	102.2 (8.2 to 214.2)	143.6 (63.8 to 297.7)	**0.0058**^c^

Comparison of the following parameters showed no significant differences among the groups: rate of patients with critical hypoglycaemia, incidence of BG values below 40 mg/dl, rate of patients with hyperglycaemia above 140 mg/dl.

^a ^Significance level p < 0.05; p values for the comparison between different therapy groups were calculated using t-test (b), Mann-Whitney-U test (c) and 2 × 2 tables (d), as appropriate.

BG = blood glucose; IQR = interquartile range; SD = standard deviation; IU = international units.

### Predisposing factors

**Figure 4:**
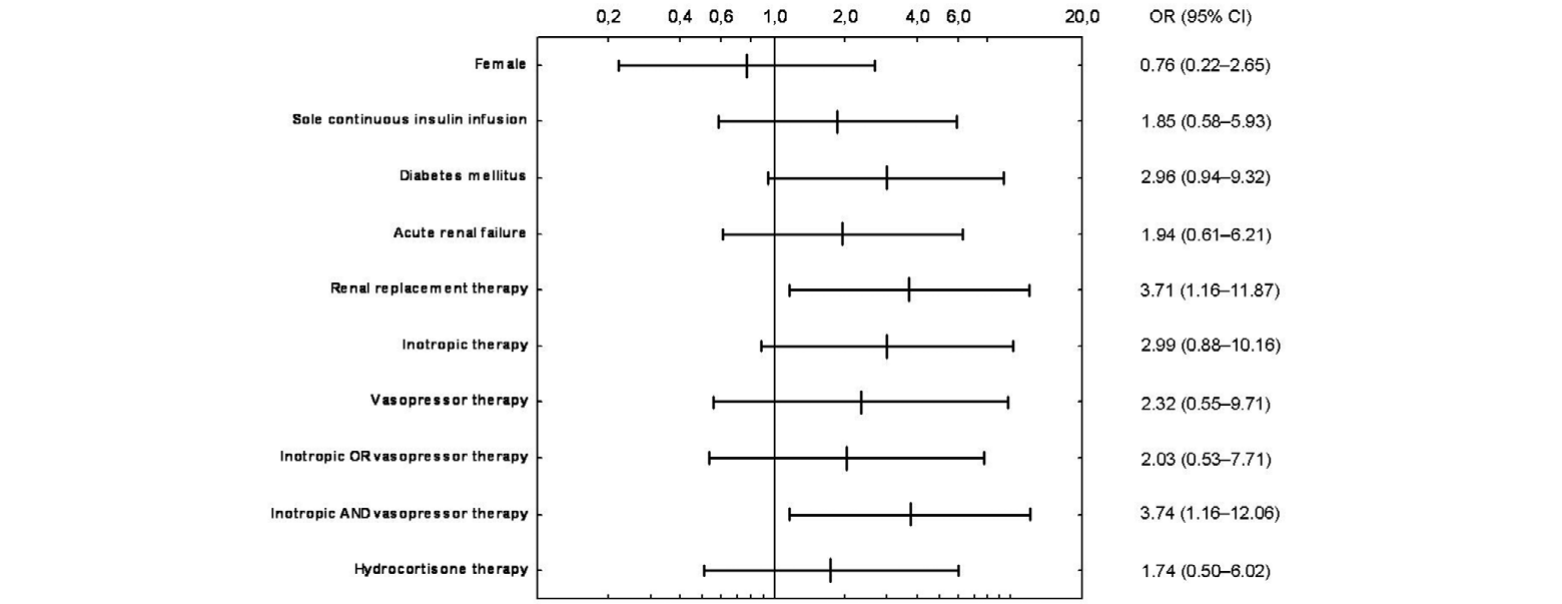
**Odds ratio and 95% confidence intervals for different potential risk factors of hypoglycaemia**. The following factors were not associated with an increased risk for hypoglycaemia: gender, age, type of admission (medical, scheduled surgical and unscheduled surgical), intensive insulin therapy (IIT) protocol, severity of illness (Simplified Acute Physiology Score (SAPS II) and Sepsis-Related Organ Failure Assessment (SOFA) scores at the first day of sepsis), acute renal failure (in contrast to continuous renal replacement therapy), vasopressor and hydrocortisone therapy, length of mechanical ventilation and length of stay.

Figure 4 summarises ORs and CIs for the analysed predisposing factors of hypoglycaemia.

### Multivariate logistic regression analyses

The SD of BG values was positively associated with critical hypoglycaemia regardless of the severity of sepsis (p = 0.0043). More specifically, the SD was significantly associated with critical hypoglycaemia only for patients with septic shock (p = 0.0197). Catecholamine therapy was not confirmed as a risk factor for critical hypoglycaemia (p = 0.2950). CRRT remained significant as a risk factor (p = 0.0476). Critical hypoglycaemia was not associated with mortality (p = 0.3211). An SD above 20 was associated with mortality (mortality rate; SD > 20 mg/dl: 24.0%, SD < 20 mg/dl: 2.5%; p = 0.0195). All patients with severe sepsis and septic shock who died had SD values above 20 mg/dl.

## Discussion

The risks and benefits of IIT are controversial. Major arguments are centred on the risk of hypoglycaemia in critically ill patients and the undefined impact on morbidity and mortality in different patient subgroups [18].

Our comparison of different sepsis levels revealed a tendency towards a higher rate of critical hypoglycaemia in patients with severe sepsis and septic shock (p = 0.1497) despite a higher upper target BG level compared with other studies. Interestingly, this effect was observed even though the median number of BG measurements per day was significantly higher for patients with septic shock. In addition, we observed a continuous increase in the rate of critical hypoglycaemia from patients with sepsis (lowest rate of hypoglycaemia) to severe sepsis to septic shock (highest rate of hypoglycaemia). This observation supports the hypothesis that, with an increased severity of illness, there is a corresponding increase in metabolic instability. Even though the increase was not significant, the occurrence of critical hypoglycaemia among 11.5% of patients with septic shock is quite high and deserves attention, especially as critical hypoglycaemia is independently associated with an increased risk of mortality [10].

In addition, a higher rate of critical hypoglycaemia among patients with severe sepsis and septic shock was reported by the results of the VISEP study from the German Sepsis Society [8]. This multicentre trial, which compared conventional insulin therapy to IIT among septic patients, was stopped prematurely because of an increased risk of critical hypoglycaemic episodes among septic patients receiving IIT. This study did not differentiate by grade and combined patients with severe sepsis and septic shock. Compared with the VISEP trial, which was conducted in surgical as well as non-surgical ICUs, our study population differs with regard to reason for admission (percentage of non-surgical patients: VISEP 46.9% versus 17.0% among our patients). Moreover the study population varies because of the different inclusion criteria (VISEP study was severe sepsis/septic shock excluding sepsis with a lesser degree of severity; mean SOFA (95% CI): VISEP 7.8 (7.4 to 8.1) versus 7.3 (6.8 to 7.7) in our patients). Using IIT, 17% of patients with severe sepsis and septic shock developed critical hypoglycaemia compared with 4.1% in the conventional treatment group [8]. The lower rate observed among our patients with either severe sepsis or septic shock (8.3%) was probably due to the higher target range utilised in our study (80 to 140 mg/dl; German Sepsis Society BG target range: 80 to 110 mg/dl). This finding was confirmed by the results of the Glucontrol trial, which compared two different target BG levels (72 to 110 mg/dl versus 141 to 180 mg/dl) for IIT in the ICU. This study was also stopped prematurely because of a higher rate of hypoglycaemia in the group randomised to the 72 to 110 mg/dl target range (publication in preparation) [18].

Other recent publications have demonstrated a higher risk of hypoglycaemia among septic patients irrespective of the severity of sepsis [11,19]. In one of these studies [19] 950 septic patients were analysed in a post hoc analysis of the surgical and medical patients from the two major Leuven studies [3,4]. The effect of IIT on outcome in patients with sepsis and a prolonged LOS was similar to the effects observed in other patients [19].

The rate of hyperglycaemia was higher in patients with severe sepsis and septic shock compared with patients with sepsis, but the rate did not differ between the group of patients with severe sepsis and the one with septic shock, even though hydrocortisone replacement therapy (200 mg/24 hours) was performed in 57.4% of patients during septic shock compared with only 4.8% of patients with severe sepsis.

Most studies of IIT have restricted their analyses to mean (morning) BG levels [3,4,8,20] and the rate of hypoglycaemic episodes [3,4,8]. Recently different studies evaluated other parameters than the mean BG level to analyse the changes of BG. They focused on glycaemic variability and its association with mortality in ICU patients showing that increasing glycaemic variability is a strong independent risk factor of mortality [21-24]. Among our patients the SD of all BG values for each patient, as an index of glycaemic variability, was significantly higher for patients with severe sepsis compared with patients with sepsis and septic shock. So no clear relation between SD and severity of sepsis was found. Further analyses showed a significant association with critical hypoglycaemia, especially for patients with septic shock. Interestingly SD levels above 20 mg/dl were associated with a significantly higher mortality rate relative to those with SD levels below 20 mg/dl (24.0% versus 2.5%).

In addition, all patients with severe sepsis and septic shock and a fatal outcome had SD levels above 20 mg/dl. So BG variability is highly associated with mortality as was also shown by the VISEP study [8]. This data suggest a positive association of the severity of disease and the variability of BG levels. Other parameters for BG variability were evaluated. Another interesting approach was introduced by Van Herpe and colleagues with the glycaemic penalty index which analyses BG variability according to the severity of BG changes [25]. Further studies with higher patient numbers are needed to evaluate the validity and reliability of different parameters for BG variability.

The following predisposing factors for hypoglycaemia were identified in this study using univariate analyses: diabetes, CRRT and inotropic combined with vasopressor therapy. In a multivariate model including several parameters, only CRRT was significantly associated with critical hypoglycaemia. Some of these predisposing factors were confirmed by other studies: diabetes, CRRT and haemodynamic shock [10,11]. Consequently, the coexistence of predisposing factors, which are also influenced by the severity of sepsis, could increase the risk of critical hypoglycaemia among these patients.

Risk factors and outcomes of critical hypoglycaemia were recently evaluated in a retrospective analysis of 102 patients with BG levels below 40 mg/dl. In this study, even a single episode of critical hypoglycaemia was independently associated with an increased risk of mortality [10].

One major limitation of our study was lack of randomisation and the significantly different rate of patients with diabetes within the different sepsis groups. Another limitation of our study was its observational study design to evaluate the implementation of an IIT protocol under real ICU conditions without the influence of the members of the study group. Also, better control of BG values with dynamic scaled IIT may have been facilitated by a learning effect of the ICU staff during the study period. To minimise this effect, an eight-week introduction period for the sliding scale protocol was performed and strict treatment protocols were used. Additionally, we did not monitor the daily calorie intake. Finally, this study is underpowered to detect differences in morbidity or mortality. Therefore, further studies are needed to confirm our results with a randomised study protocol. Further research will permit analysis of additional parameters to evaluate BG profiles and BG variability, which will improve the quality of studies on IIT. In addition, identification of additional predisposing factors for hypoglycaemia, evaluation of the consequences of hypoglycaemia in ICU patients and determination of the effects of IIT on morbidity and mortality among these patients can be undertaken in further studies.

## Conclusion

Patients with severe sepsis and septic shock treated with IIT have a high risk for hypoglycaemia and hyperglycaemia. Our results confirm those of other studies showing a higher risk of hypoglycaemia using IIT, even though we used a higher target range of 80 to 140 mg/dl. Therefore an increased awareness during IIT in patients with severe sepsis and septic shock is mandatory in order to enhance patient safety. A higher rate of BG measurements would be indicated in these patients but might be difficult to achieve outside controlled studies as long as continuous measurements are not available. Glycaemic variability, reflected by the SD of the mean BG level for each patient, was independently associated with both risk of severe hypoglycaemia and the risk of mortality.

## Key messages

• The risks and benefits of IIT are controversial; more specifically the risk of hypoglycaemia and its undefined impact on morbidity and mortality in patients with severe sepsis and septic shock are debated.

• Patients with severe sepsis and septic shock treated with IIT have a high incidence of critical hypoglycaemia, even with a higher target BG range of 80 and 140 mg/dl.

• Glycaemic variability, reflected by the SD of the mean BG level for each patient, was independently associated with both the risk of severe hypoglycaemia and the risk of mortality.

• Predisposing factors for critical hypoglycaemia were continuous renal therapy and combined inotropic and vasopressor therapy.

• Increased awareness for critical hypoglycaemia during IIT of patients with severe sepsis and septic shock is mandatory in order to enhance patient safety.

## Abbreviations

ANOVA: analysis of variance; BG: blood glucose; BMI: body mass index; CI: confidence interval; CRRT: continuous renal replacement therapy; GCS: Glasgow Coma Scale; ICU: intensive care unit; IIT: intensive insulin therapy; IQR: interquartile range; LOS: length of stay; OR: odds ratio; PDMS: patient data management system; SD: standard deviation; SAPS: Simplified Acute Physiology Score; SIRS: systemic inflammatory response syndrome; SOFA: Sepsis-Related Organ Failure Assessment; VISEP: Efficacy of Volume Substitution and Insulin Therapy in Severe Sepsis; WBC: white blood cells.

## Competing interests

The authors declare that they have no competing interests.

## Authors' contributions

RW participated in the conception and design of the study, carried out the data collection and data analysis, and drafted the manuscript. OM participated in the conception and design of the study and drafting of the manuscript. RH performed the statistical analysis and participated in the drafting of the manuscript. PH participated in the programming data analysis. PN participated in the design of the study and drafting of the manuscript. MQ conceived the study, participated in its design, was involved in its coordination, and helped to draft and revise the manuscript.

## Supplementary Material

Additional file 1 A pdf file containing a table that shows protocol 1 (sliding scale) and protocol 2 (dynamic scale) used for IIT. Click here for file

Additional file 2 A pdf file containing a figure showing the mean BG level per day over the time for the two therapy protocols used. Each column represents the mean BG level of all patients at the first 28 days after admission. The numbers above the error bars indicate the number of patients involved. Click here for file
