# Supplementary material for: The impact of the severity of sepsis on the risk of hypoglycaemia and glycaemic variability
Source: Crit Care. 2008 Oct 21;12(5):R129. doi: 10.1186/cc7097 (PMC2592768; doi:10.1186/cc7097)
Supplement: Additional file 1 — A pdf file containing a table that shows protocol 1 (sliding scale) and protocol 2 (dynamic scale) used for IIT. [file cc7097-S1.pdf]

Protocol 1: Sliding scale protocol with a combination of continuous insulin infusion and bolus application

Basic therapy

Blood glucose (mg/dl)	Adjustment of insulin infusion rate
< 50	Stop insulin infusion, check glucose, call MD, 40ml glucose 40% bolus, recheck blood glucose after 10 min
50 – 60	Stop insulin infusion, check glucose, call MD, continuous glucose infusion, recheck blood glucose after 60 min
60 – 100	No Changes
100 – 140	Reduction of insulin infusion according to the experience with the patient, in doubt call MD and stop insulin
140 – 170	Insulin infusion 2 U/h

Following insulin therapy adjustments (do not use for initial therapy)

Blood glucose (mg/dl)	Adjustment of insulin infusion rate
170 – 200	Insulin infusion 4 U/h + 3 U bolus application
200 – 230	Insulin infusion 4 U/h + 6 U bolus application
230 – 260	Insulin infusion 4 U/h + 9 U bolus application
260 – 300	Insulin infusion 4 U/h + 12 U bolus application
> 300	Insulin infusion 4 U/h + 15 U bolus application

- If blood glucose level is not falling after repeating controls, call MD for advance therapy on the next level
- Advanced attention if changes in body temperature > 1° C and medication are noticed
- If no other order is given, check blood glucose level every 3 hours
- If blood glucose level is more than 50 % reduced, check level in 60 min again

Protocol 2: Dynamic scale protocol with isolated continuous insulin infusion

Initial infusion rate

Blood glucose (mg/dl)	< 110	110 – 140	141 – 180	> 180
Insulin infusion rate (units/h)	0	1	2	3

Following infusion rate

Blood glucose (mg/dl)	Adjustment of insulin infusion rate
< 50	Stop insulin infusion, check glucose, call MD, 40ml glucose 40% bolus, recheck blood glucose after 10 min
50 - 59	Stop insulin infusion, check glucose, call MD, continuous glucose infusion, recheck blood glucose after 60 min
60 – 79	If current rate is > 5 U/h, reduce rate by 2 U/h<br>If current rate is ≤ 5 U/h, reduce rate by 0.5 U/h
80 – 109	No changes, if rate is ≥ 4 U/h, check blood glucose hourly
110 – 140	If blood glucose is lower than at last control, no changes<br>If blood glucose is higher than at last control, increase rate by 1-2 U/h
141 – 179	If blood glucose is lower than at last control, no changes<br>If blood glucose is higher than at last control, increase rate by 1-2 U/h
180 – 219	If blood glucose is lower than at last control, no changes<br>If blood glucose is higher than at last control, increase rate by 2 U/h
220 – 300 / 12.2 – 16.7	If blood glucose is lower than at last control, no changes<br>If blood glucose is higher than at last control, increase rate by 3 U/h
> 300	Increase rate by 3-4 U/h, if blood glucose is > 300 mg/dl over 3 controls, increase rate by 50%, call MD, check blood glucose after 30 min

- If no other order is given, check blood glucose level every 3 hours
- Insulin application solely continuous, no bolus
- If blood glucose is reduced by more than 50 %, reduce insulin infusion rate by 50 % as well and call MD. Check blood glucose after 1 h
- If the blood glucose level does not reduced under 9.9 mmol/L within 8 h, call MD
- Minimal infusion rate in diabetes type I patients 0.5 U/h
