# Supplementary figures and images for: The impact of the severity of sepsis on the risk of hypoglycaemia and glycaemic variability
Source: Crit Care. 2008 Oct 21;12(5):R129. doi: 10.1186/cc7097 (PMC2592768; doi:10.1186/cc7097)

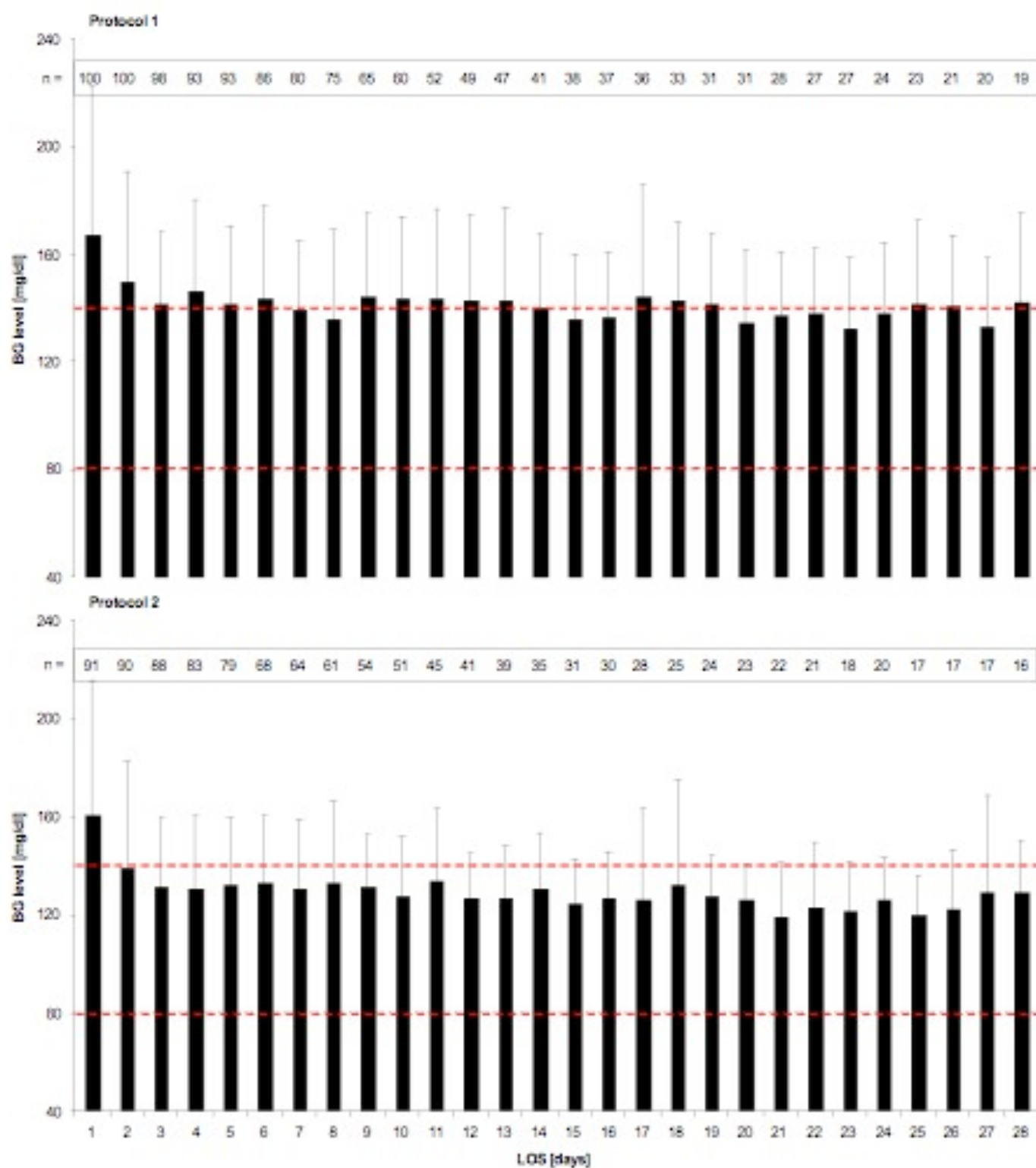

Supplement: Additional file 2 — A pdf file containing a figure showing the mean BG level per day over the time for the two therapy protocols used. Each column represents the mean BG level of all patients at the first 28 days after admission. The numbers above the error bars indicate the number of patients involved. [file cc7097-S2.pdf]
